# Current Status on Marine Products with Reversal Effect on Cancer Multidrug Resistance

**DOI:** 10.3390/md10102312

**Published:** 2012-10-19

**Authors:** Ioana Abraham, Khalid El Sayed, Zhe-Sheng Chen, Huiqin Guo

**Affiliations:** 1 Department of Pharmaceutical Sciences, College of Pharmacy and Health Sciences, St. John’s University, Queens, NY 11439, USA; Email: ioana.abraham@yahoo.com; 2 Department of Basic Pharmaceutical Sciences, College of Pharmacy, University of Louisiana at Monroe, Monroe, LA 71201, USA; Email: elsayed@ulm.edu; 3 Department of Thoracic Surgery, Peking Union Medical College Hospital, Beijing 100730, China

**Keywords:** marine natural products, multidrug resistance, ABC transporters

## Abstract

The resistance of tumor cells to a broad range of anticancer agents continues to be a problem for the success of cancer chemotherapy. Multidrug resistance (MDR) is due in part to three drug transporter proteins: ABCB1/P-glycoprotein (P-gp), ABCC1/multidrug resistance protein 1 (MRP1) and ABCG2/breast cancer resistance protein (BCRP). These transporters are part of the ATP-binding cassette (ABC) superfamily, whose members function as ATP-dependent drug-efflux pumps. Their activity can be blocked by various drugs such as verapamil (calcium channel blocker) and cyclosporin A (immunosuppressive agent), *etc*. These compounds are called MDR modulators or reversals. This review highlights several marine natural products with reversal effect on multidrug resistance in cancer, including agosterol A, ecteinascidin 743, sipholane triterpenoids, bryostatin 1, and welwitindolinones.

## 1. Introduction

The ocean, which covers around 70% of the Earth’s surface, is a rich source of natural resources that contains nearly 80% of all the varieties of life on our planet. During the past 30 years, thousands of new compounds with different biological activities varying from anticancer to antiviral have been isolated from marine sources [1,2,3]. Currently, over 20,000 natural marine products are isolated and identified from various organisms, including sponge, algae, coral and ascidian [4]. Ziconotide (Prialt; Elan Pharmaceuticals), a peptide from a tropical cone snail, was the 3rd natural product approved in the US in 2004 for pain treatment [5]. Cytarabine (Cytosar-U, Ara-C, Depocyt; Bedford Laboratories-Enzon Pharmaceuticals) and vidarabine (Ara-A, Vira-A; King Pharmaceuticals) were sponge-derived drugs FDA-approved for cancer and viral infections, respectively [5,6,7]. Approximately 150 compounds are considered to be cytotoxic against tumor cells and at least a dozen of them are in various phases of clinical trials for the treatment of cancer [1,8]. In 2007, ecteinascidin 743 (trabectedin, brand name Yondelis; PharmaMar) was approved in the European Union for advanced soft tissue sarcoma [9,10,11,12]. The macrolide eribulin mesylate and the peptide soblidotin are also marine-derived compounds currently in phase III clinical trials for cancer [5,13,14]. Ten other marine natural products are in clinical trials phases I/II clinical trials including synthadotin (ILX651) [15,16], dolatostatin-10 [17,18], bryostatin-1 [19], and aplidin [20].

One of the major problems associated with cancer chemotherapy is resistance to anticancer drugs. Some cancers such as rectal and lung cancer show natural resistance, or primary resistance, in which they are not initially sensitive to standard chemotherapy. Meanwhile, many types of tumors respond well to chemotherapy at the beginning but gradually develop acquired resistance later. Resistance can occur in response to particular cytotoxic drugs, but can also occur to various drugs with different chemical structures and mechanisms of action. This later form of resistance is called multidrug resistance (MDR) [21]. There are a few mechanisms of MDR in tumor cells: Decreased uptake of water-soluble drugs (e.g., folate antagonists, nucleoside analogs and cisplatin) that require transporters to enter cells, changes in cells that decrease the ability of cytotoxic drugs to kill cells (*i.e*., alterations in cell cycle, defective apoptotic pathways and altered drug metabolism), and increased energy-dependent efflux of hydrophobic drugs that go through the plasma membrane by diffusion [22]. An important mechanism of MDR is increased efflux mediated by ATP-binding cassette (ABC) transporters of drugs out of cells. These transporters use the energy that is released when ATP is hydrolyzed to transport molecules across the cell membrane. The most significant efflux pumps found to confer chemoresistance in cancer are members of the ABC transporter family, such as ABCB1/P-glycoprotein (P-gp)/Multidrug resistance 1 (MDR1), ABCC1/multidrug resistance protein 1 (MRP1), and ABCG2/breast cancer resistance protein (BCRP) [23]. 

**Figure 1:**
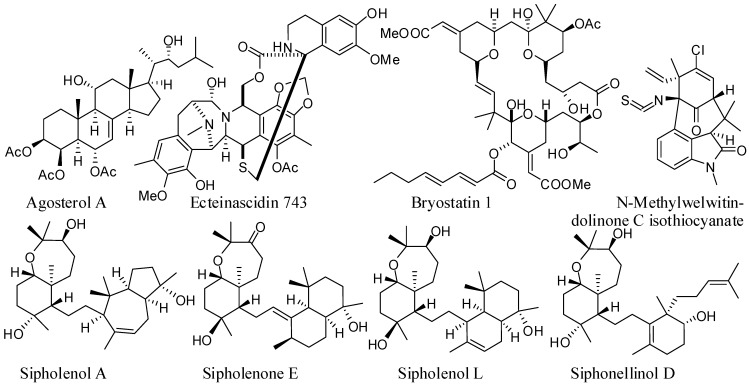
Structure of marine natural products with reversal effect on multidrug resistance in cancer cells.

Once MDR is developed, chemotherapy is no longer effective, even with the use of high doses of drugs to overcome resistance. This problem can be resolved by simultaneously administering anticancer drugs with ABC transporter inhibitors. These ABC transporter inhibitors, also called MDR modulators, chemosensitizers, or MDR reversal agents, are able to reverse resistance against anticancer drugs [24,25,26,27]. Even though, better results have been observed in cell culture then in pre-clinical settings, significant efforts have been made in search for new entities useful as MDR modulators. These efforts resulted in the identification of a number of marine compounds that are able to reverse MDR, such as agosterol A, ecteinascidin 743, sipholane triterpenoids, bryostatin 1, and welwintolidones (Table 1, Figure 1). This review highlights these marine compounds, their activity as MDR reversals, and their impact on the chemotherapy of various malignancies.

**Table 1:** Marine products with reversal effect on multidrug resistance in cancer cells.

Compounds	Source organism	Chemical class	Cell lines used	Refences
Agosterol A (AG-A)	*Spongia* sp.	polyhydroxylated sterol acetate	P-gp/MDR1-overexpressing (KB-C2)	[28,29,30]
			MRP1-overexpressing (KB-CV60)	[28,29,30]
			MRP1-transfected (KB/MRP)	[31]
Ecteinascidin 743 (ET-743)	*Ecteinascidia turbinate*	tetrahydroiso-quinolone alkaloid	P-gp/MDR1-overexpressing (KB-8-5, KB-C2)	[32]
Sipholenol A	*Callyspongia siphonella*	sipholane triterpenoids	P-gp/MDR1-overexpressing (KB-C2, KB-V1)	[33,34,35]
Sipholenone E Sipholenol L Siphonellinol D			P-gp/MDR1-overexpressing (KB-C2)	[36]
Bryostatin 1	*Bugula neritina*	macrocyclic lactone	P-gp/MDR1-overexpressing (KB-C1, HeLa-MDR1-V185)	[37]
*N-*Methylwelwitin-dolinone C isothiocyanate	*Hapalosiphon welwitschii*	alkaloid	P-gp/MDR1-overexpressing (SK-VLB-1, MCF-7/ADR)	[38]

Abbreviations: P-glycoprotein (P-gp)/Multidrug resistance 1 (MDR1), Multidrug resistance protein 1 (MRP1).

## 2. Agosterol A (AG-A)

Agosterol A is a polyhydroxylated sterol acetate which has been isolated from the marine sponge *Spongia* sp. [28]. The ethyl acetate-soluble portion of the acetone extract of *Spongia* sp. collected at Ago Bay, Mie Prefecture in Japan, showed growth inhibition of P-gp overexpressing MDR tumor cells (KB-C2) in the presence of 0.1 μg/mL of colchicine, while it presented no cytotoxicity against parental KB-3-1 cells at the same concentration [28]. The bio-assay guided separation identified the most active compound to be agosterol A [28]. Further investigation of the same sponge extract led to the isolation of the sterol analogues agosterols B, C, A_4_, D_2_, A_5_ and C_6_[29]. Agosterol A showed the strongest activity by completely reversing the resistance against colchicine in KB-C2 and vincristine in KB-CV60 cells, at 1 μg/mL concentration. Even at 10 μg/mL concentration, agosterol A was not cytotoxic to parental cells. Subsequent biological evaluation of agosterol A was published two years later in the Japanese Journal of Cancer Research [30]. Agosterol A completely reversed the resistance to vincristine, colchicine, doxorubicin, and etoposide in both KB-C2 and KB-CV60 cells at 3 μM concentration. To investigate the mechanism of agosterol A overcoming the resistance to vincristine in these two cell lines, accumulation and efflux studies were performed. Agosterol A inhibited the ATP-dependent active efflux of vincristine in both KB-C2 and KB-CV60 cells by increasing intracellular vincristine. Moreover, AG-A inhibited both the [^3^*H*]azidopine-photolabeling of P-gp and the uptake of [^3^*H*]*S*-(2,4-dinitrophenyl)glutathione (DNP-SG) in inside-out membrane vesicles from KB-CV60 cells. This data indicated that AG-A inhibited drug efflux through P-gp and MRP1. In the same year, another paper was published on the mechanism of AG-A’s action [31]. Furthermore, its potency was compared to known MRP1 inhibitors. AG-A inhibited MRP1-mediated transport of typical amphipathic substrates, increased the drug accumulation of vincristine in MRP1-transfected cells, and reduced intracellular glutathione levels [31]. This suggests that AG-A diminishes MRP1-mediated drug resistance by both directly inhibiting the ability of the pump to transport drugs and reducing the levels of the cellular component glutathione required for drug efflux.

## 3. Ecteinascidin 743 (ET-743)

Investigations of marine organisms with anticancer-type activity showed that aqueous extracts of the Caribbean tunicate *Ecteinascidia turbinata* contain potent ingredients. Ecteinascidins were determined to be tetrahydroisoquinolone alkaloids [39,40], with ecteinascidin-743 (ET-743) being the major metabolite. Due to its potent *in vitro* cytotoxicity (IC_50_ 0.5 ng/mL versus L1210 leukemia cells), stability, and high natural abundance yield, ET-743 was quickly promoted from hit to lead and finally became a drug candidate appropriate for clinical development. In order to perform basic studies for the mechanism of action and preclinical *in vivo* studies, large amounts of the tunicate had to be collected. Currently, ET-743 is obtained by a semisynthetic process using cyanosafracin B obtained in bulk through fermentation of the marine bacterium *Pseudomonas fluorescens* [9]. ET-743 has been reported to bind to the minor groove of DNA, thus bending the DNA helix towards the major groove [32]. Its mechanism of action also includes interference with cellular transcription-coupled nucleotide excision repair to induce cell death and cytotoxicity [41].

Overcoming multidrug resistance in P-glycoprotein/MDR1-overexpressing cell lines by ET-743 was investigated by Kanzaki *et al.* [42]. Studies using KB-8-5 and KB-C2 cells overexpressing P-gp/MDR1 demonstrated that a nontoxic concentration of ET-743 partially reversed the resistance to both doxorubicin (DOX) and vincristine (VCR). Pretreatment with ET-743 increased the accumulation of these two drugs, probably by down-regulating expression of P-gp. Moreover, overcoming of DOX/VCR resistance was not due to the direct inhibition of P-gp activity, as determined by the photoaffinity labeling experiments. This data suggests that combination of ET-743 with chemotherapeutic agents that are substrates for P-gp/MDR1 may be used in the clinic.

In general, advanced breast, ovarian, and mesenchymal tumors which have been previously treated with platinum and taxanes showed the best response to ET-743 in phase I trials [43]. In phase II trials, ET-743 was most effective in patients with refractory soft tissue sarcoma (STS), breast, and ovarian cancer. ET-743 has received orphan drug status for STS in the United States and in ovarian cancer in patients with recurrent ovarian cancer in both the United States and Europe [44].

## 4. Sipholane Triterpenoids

The sipholane triterpenoids were isolated from the Red Sea sponge *Callyspongia siphonella*. They possess a perhydrobenzoxepine (rings “A” and “B”) and a [5,3,0]bicyclodecane system (rings “C” and “D”), linked together through an ethylene bridge [33,34]. Thus far, 30 triterpenoids have been isolated from this sponge, consisting of four different skeletons: sipholane, siphonellane, neviotane and dahabane [33,34]. Among these four types, sipholane triterpenoids are the most important, and include sipholenol A, sipholenone E, sipholenol L and siphonellinol D. Sipholenol A has been reported by Shi Z *et al.* to reverse P-glycoprotein (ABCB1)-mediated MDR in cancer cells [35]. Sipholenol A increased the cytotoxicity of known P-gp substrate anticancer drugs such as colchicine, vinblastine, and paclitaxel, and reversed the MDR of KB-C2 and KB-V1 cells. It did not alter the sensitivity of vincristine and mitoxantrone in MRP1- and BCRP-overexpressing MDR cancer cells, respectively. Moreover, this marine natural product increased the accumulation of paclitaxel by directly inhibiting P-gp-mediated drug efflux, stimulated ATPase activity, and inhibited the photolabeling of P-gp with its transport substrate [^125^I]-iodoarylazidoprazosin. Treatment of KB-C2 and KB-V1 cells with sipholenol A for 36 and 72 h had no effect on P-gp expression. These data indicate that sipholenol A inhibited the function of P-gp through direct interactions and attested the potential of sipholane triterpenoids as a new class of P-gp reversing agents [35]. This prompted more investigation of related sipholane triterpenoids from the same sponge. Sipholenone E, sipholenol L and siphonellinol D were later found to inhibit the function of P-gp [36]. They enhanced the cytotoxicity of several P-gp substrate anticancer drugs and reversed the MDR-phenotype in KB-C2 cells in a similar fashion to sipholenol A. These sipholanes had no effect on the response to cytotoxic agents in MRP1-, MRP7- and BCRP-overexpressing cells. They increased the accumulation of [3H]-paclitaxel and calcein by inhibiting the drug efflux function of P-gp. All three triterpenoids stimulated P-gp ATPase activity and inhibited the photolabeling of this transporter with IAAP, suggesting that they directly interact with P-gp. In silico molecular docking analysis identified the ligand binding sites of these compounds. In conclusion, sipholenone E, sipholenol L and siphonellinol D, like sipholenol A, represent potential reversal agents for the treatment of MDR in P-gp-overexpressing tumors.

## 5. Bryostatin 1

Bryostatin 1, a macrocyclic lactone isolated from the marine bryozoan *Bugula neritina*, is an antitumor agent which modulates the enzyme activity of protein kinase C (PKC, phospholipid-Ca^2+^-dependent ATP: protein transferase, EC 2.7.1.37) [45]. The activity of bryostatin 1 is similar to that of the tumor-promoting phorbol ester, TPA (12-*O*-tetradecanoylphorbol-13-acetate), since both agents activate PKC upon binding [46,47]. Extended exposure of intact cells to either bryostatin 1 or TPA induces the translocation of PKC from the cytosol to the membrane and a depletion of the enzyme [48]. 

Several reports have suggested that the pumping activity of multidrug transporter P-glycoprotein is enhanced by a PKC-mediated phosphorylation [49]. Bryostatin 1, in addition to having antitumor activity, was also able to modulate P-gp mediated MDR. Spitaler *et al.* reported the ability of bryostatin 1 to reverse the resistance to vinblastine and colchicine in two cell lines over-expressing a mutant MDR1-encoded P-gp: KB-C1 and HeLa cells transfected with an MDR1-V185 construct (HeLa-MDR1-V185) in which glycine at position 185 (G185) was substituted by valine (V185) [37]. Bryostatin 1 did not reverse the resistance of cells over-expressing the wild-type form (G185) of P-gp (*i.e.*, CCRF-ADR5000 and HeLa-MDR1-G185 cells). Treatment of the HeLa-MDR1-V185 cells with bryostatin 1 led to an increase in the intracellular accumulation of rhodamine 123, while no effect was observed in the wild type form (G185). Reversal of P-gp mediated MDR by bryostatin 1 was not mediated by a PKC-dependent mechanism but rather by a mutation at position 185 of P-gp. Moreover, bryostatin 1 binds G185 as well as V185 sites of P-gp. These results show that this agent was able to reverse a specific mutant P-gp. 

## 6. Welwitindolinones

Welwitindolinones, a family of alkaloids isolated from the blue-green alga *Hapalosiphon welwitschii*,were reported by Smith *et al.* to reverse P-glycoprotein MDR [38]. The pharmacological activities of the three structurally related members of this family were compared. *N*-Methylwelwitindolinone C isothiocyanate had reversing efficacy similar to that of verapamil in two different MDR cell lines. *N*-Methylwelwitindolinone C increased the cytotoxicity of actinomycin D and daunomycin in SK-VLB-1 cells. It also decreased the IC_50_ values of vinblastine, taxol, actinomycin D, colchicine and daunomycin in the drug-resistant breast carcinoma (MCF-7/ADR) cells. While welwitindolinone C isothiocyanate exhibited a weaker reversing activity, an analogue of the former compound with the isothiocyanate group replaced by an isonitrile group was inactive. *N*-Methylwelwitindolinone C isothiocyanate proved to be the most potent derivative by its ability to increase the accumulation of [^3^*H*]-vinblastine and [^3^*H*]-paclitaxel as well as inhibit the P-glycoprotein photoaffinity labeling by [^3^*H*]-azidopine in MDR cells. As a result, replacement of the isothiocyanate by the isonitrile group seems to adversely influence the activity and ability of these agents to interact with P-gp. 

## 7. Conclusions

The development of anticancer marine natural products is one of the most important approaches of global drug discovery due to the fact that the marine ecosystem has an abundant number of species and unique chemical diversity. In recent decades, countless numbers of marine compounds and related analogues have been discovered, and many of them have shown reversing activity in MDR cancer cells. Such agents represent novel scaffolds for the discovery and development of effective P-gp modulators, not only for their potential to be used in combination with chemotherapy treatment, but also to rationally design analogues with higher potency and fewer pharmacokinetic interactions, especially with the recent availability of the P-gp crystal structure. 
